# Lipid-Based Nanocarriers for Topical Therapy of Cutaneous Leishmaniasis: An Insight into the Mechanism of Action

**DOI:** 10.1021/acsomega.5c00046

**Published:** 2025-06-05

**Authors:** Amina Riaz, Muhammad Farhan Ali Khan, Aamir Jalil, Asim ur Rehman, Naveed Ahmed, Zobia Mubarak, Maryam Parhizkar, Hira Muqaddas

**Affiliations:** a Department of Pharmacy, 389430 The Women University Multan, Multan 6000, Pakistan; b School of Pharmacy, 4919 University College London, London WC1N 1AX, United Kingdom; c Department of Pharmaceutics, Faculty of Pharmacy, Bahauddin Zakariya University, Multan 60800, Pakistan; d Department of Pharmacy, Faculty of Biological Sciences, 66757 Quaid-i-Azam University, Islamabad 45320, Pakistan; e Punjab University College of Pharmacy, University of the Punjab, Lahore 54000, Pakistan; f Primary and Secondary health care department, Govt of Punjab, Lahore 54000, Pakistan; g Department of Zoology, 389430 The Women University Multan, Multan 6000, Pakistan

## Abstract

Cutaneous leishmaniasis (CL) is a parasitic infection caused by Leishmania species, affecting millions worldwide. The current treatment options for CL have several limitations, including low efficacy, inability to reach the target site, potential toxicity, and longer treatment duration, leading to poor patient compliance. Therefore, there is an urgent need for alternative treatments, especially topical ones, that are more effective, safe, and patient-friendly. Although numerous reviews have described the advantages of a wide range of nanoparticles in the treatment of CL, this review narrows its focus to the utilization of novel lipid-based nanocarriers for the topical treatment of CL, offering an in-depth analysis of the topical potential and mechanism of skin permeation of these lipidic nanocarriers. Lipid-based nanostructures such as liposomes, solid lipid nanoparticles, and nanostructured lipid carriers have been extensively studied for CL treatment, either alone or in combination with other drugs or therapies. These carriers can improve the bioavailability, stability, and efficacy of the drug, target the infected site, and reduce adverse effects on healthy tissues. Moreover, these can be easily formulated into different dosage forms, such as creams, gels, or ointments, for convenient topical application. Despite the many benefits of lipid-based carriers, there are still some challenges that need to be addressed, such as optimizing the formulation parameters, ensuring the reproducibility and scalability of the process, and evaluating the long-term safety and efficacy in clinical trials. This study aims to provide a comprehensive overview of the current state-of-the-art lipid-based nanocarriers for topical treatment of CL, covering the recent advances, limitations, clinical evidence, and prospects of this promising approach. In addition, the skin and macrophage targeting potential of various lipid-based nanocarriers is also discussed, which is especially helpful in treating the lesions of CL.

## Graphical abstract

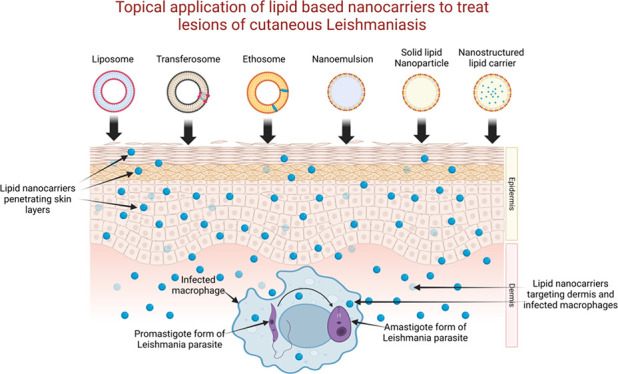

## Introduction

### Overview of Leishmaniasis

Leishmaniasis is a term used for the group of diseases that are caused by the bite of phlebotomine sandflies. The disease typically shows three types of patterns in infected individuals, i.e., cutaneous, mucocutaneous, and visceral Leishmaniasis.^1^ Cutaneous Leishmaniasis, the most common form of the disease, is categorized into localized cutaneous Leishmaniasis (LCL), diffuse cutaneous Leishmaniasis (DCL), and mucocutaneous Leishmaniasis (MCL) depending on the area of the body involved. Visceral Leishmaniasis involves the transfer of parasites into the vital organs and is the most severe form of the disease.^2^ Cutaneous Leishmaniasis is an endemic disease in more than 70 countries of the world (1.5 million new cases each year), with 90% of cases reported in Pakistan, India, Ethiopia, Brazil, Afghanistan, Peru, and Saudi Arabia.^3^ The reported number of cases, especially coinfection with human immunodeficiency virus (HIV), is increasing globally each year. According to WHO, up to 70% of cases of VL are reported in adults having coinfection with HIV in southern Europe.^4^

### Life Cycle and Clinical Symptoms of Cutaneous Leishmaniasis

When an infected sand fly bites the host, it transfers promastigotes into the skin, which are phagocytosed by host cells, mainly macrophages. Once inside the phagolysosomes of macrophages, promastigotes develop into amastigotes, which infect further macrophages present locally or at distant sites. The leishmania cycle is completed when an otherwise healthy sand fly bites an infected host, leading to the transfer of amastigotes. These amastigotes subsequently transform into promastigotes in the sand fly’s gut and the parasite is ultimately migrated into proboscis.^5^ Figure 1 represents the stages of the lifecycle of the *Leishmania* parasite.

**Figure 1:**
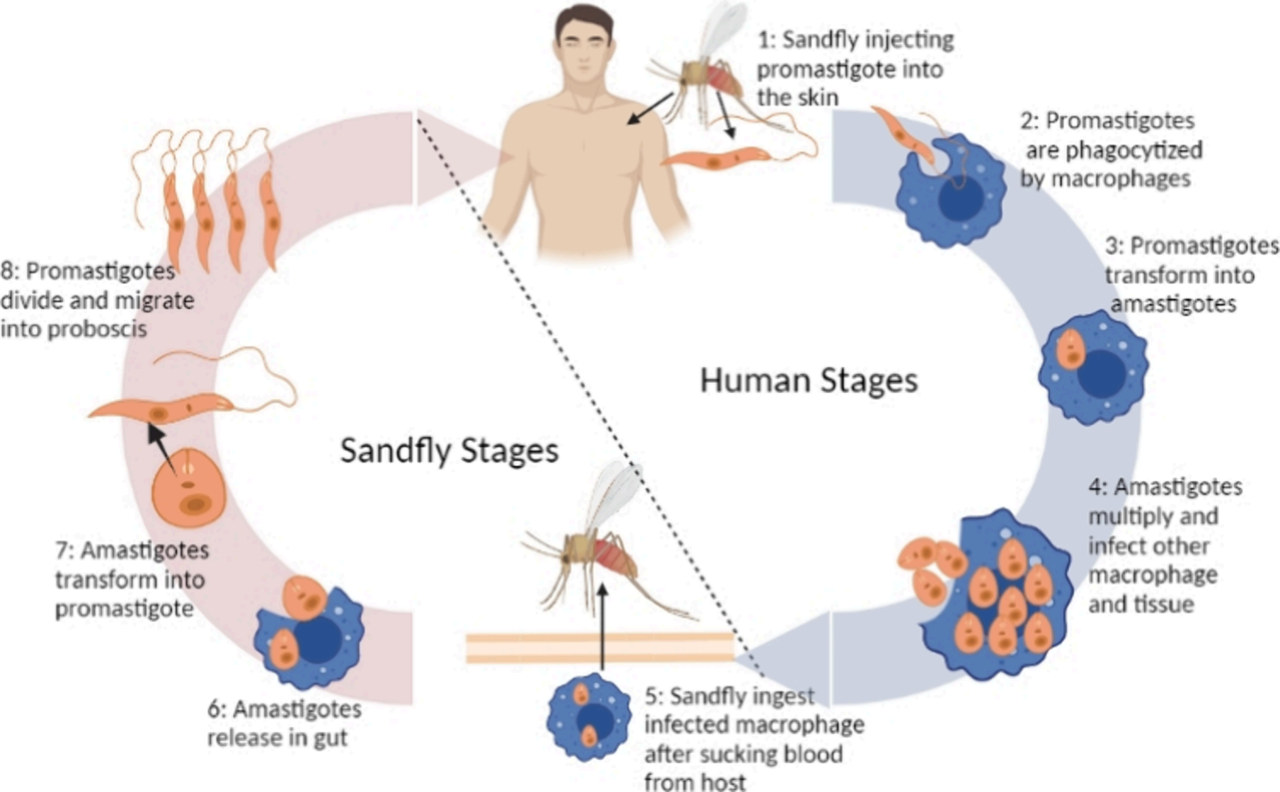
Life cycle of *Leishmania* parasites (created in BioRender; Muqaddas, H. (2025) https://BioRender.com/yh568bs).

Most infections with Leishmania species remain symptomless,^6^ and where the symptoms appear, the first sign is usually erythema at the site of the bite. The characteristic lesion of LCL is developed after erythema develops into a nodule and then a papule, which ulcerates over 2 weeks to 6 months.^7^ These lesions vary in size, appearance, and the time they take to cure spontaneously. Time to cure depends on the species of Leishmania involved, e.g., in the case of L. major, the lesion takes 2–6 months to heal,^8^ whereas in the case of L. mexicanaand L. tropica *,* they take 3–9 and 6–15 months, respectively.^9,10^ Figure 2 represents various changes that may occur in the skin throughout the disease, including ulcerative and scaly lesions.

**Figure 2:**
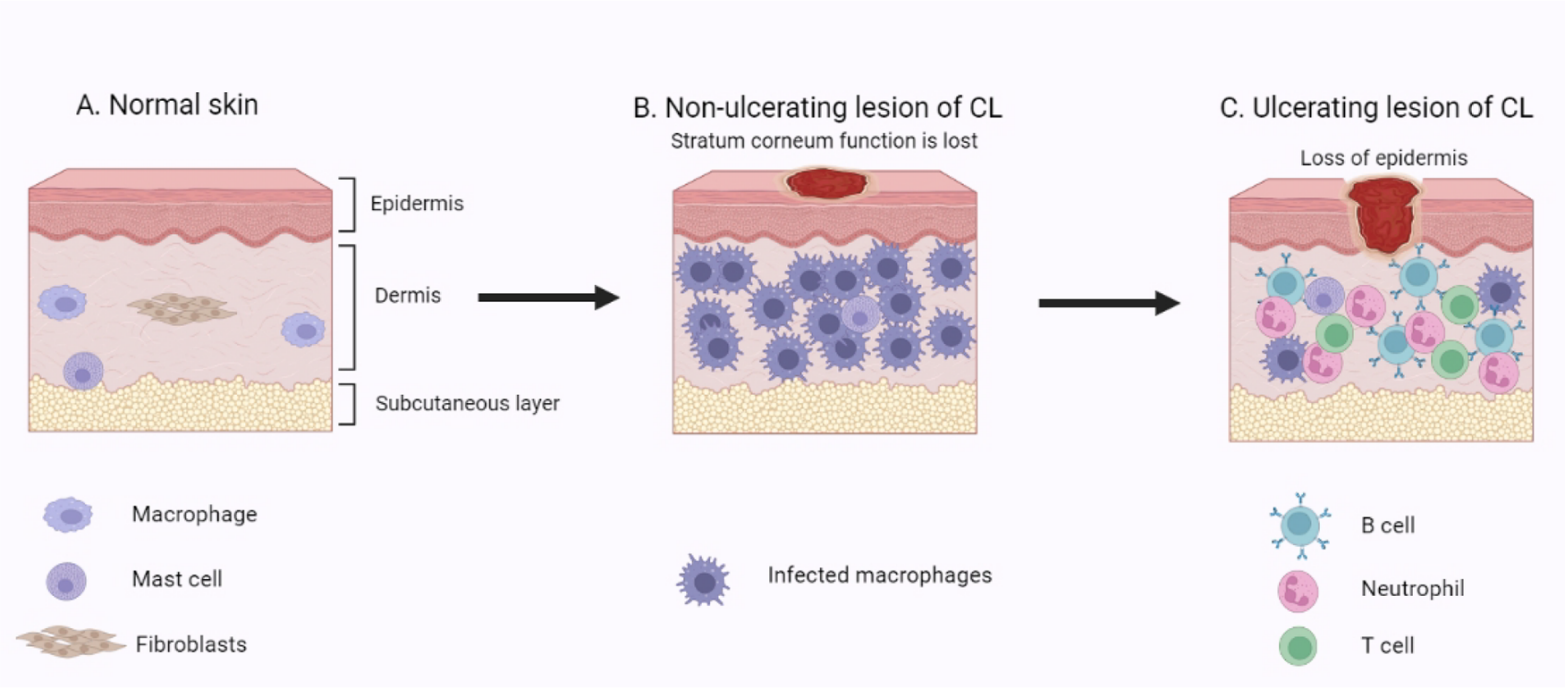
Various skin changes may occur during the course of the disease of CL. (A) Intact skin was treated with stratum corneum, viable epidermis, and dermis. (B) Lesion characterized by partial damage to the skin, stratum corneum barrier function is lost, and dermis filled with infected macrophages. (C) Ulcerative lesion characterized by loss of stratum corneum and epidermis, fewer macrophages, and increased inflammatory mediators (created in BioRender; Riaz, A. (2025) https://BioRender.com/in2fttn).

### Current Treatment and Hurdles/Challenges

In addition to intrinsic activity, the arrival of active drug ingredients at the site of action and therapeutic concentration is equally important for efficacy. In the case of cutaneous Leishmaniasis, the target is infected macrophages that reside in the deeper layer of skin, i.e., dermis. The main reason for the insufficient efficacy of topical treatment is the difficulty for active ingredients to cross the stratum corneum barrier of the skin and to overcome rapid systemic clearance once they reach the dermis. Further, various skin lesion patterns ranging from nodules and scales to ulcers throughout the disease also significantly affect drug absorption from topical sites.^11^ Therefore, the design of intelligent new formulations and the discovery of new potential targets are required to overcome the aforementioned problems.

Table 1 shows various treatment options for CL, which include parenteral, oral, intralesional, and topical therapy. Pentavalent antimonials, including sodium stibogluconate and meglumine antimoniate, are the first-line treatments for any form of Leishmaniasis. They were given parenterally as well as in the form of intralesional injections. However, research data shows no significant difference in the efficacy of one over the other.^12^ Other drugs that are being used include Amphotericin B and its liposomal formulation Ambisome, paromomycin, miltefosine, buparvaquone, and the immunomodulator drug imiquimod.^13^ Several repurposed drugs, including azole antifungals, antiparasitic, anticancer, antibiotics, and antihypertensives, were found to be effective against the promastigotes and/or amastigotes of various species of *Leishmania*. These drugs and potential targets for drug repurposing are an extensive research area and have been reviewed in detail previously. The readers are referred to these reviews for further study on the repurposing of drugs to discover potential new drugs with antileishmanial activities.^14,15^ In addition, several herbal systems and oils derived from plants were found to be effective against various *Leishmania* species *in vitro* and *in vivo*.^16^ One such oil, Annatto oil, derived from the plant Bixa orellana has demonstrated the antileishmanial potential *in vitro* and diseased animal models when administered intraperitoneally.^17^ Various medicinal plants, including Almond, Thyme, Aloe, Garlic, and Marigold, were scientifically proven to be effective in treating lesions of CL and reducing the parasite burden.^18^ The uncomplicated cases may be treated without requiring hospital admission, but frequent visits to health care centers are required. However, these therapies are far from being satisfactory because of the emergence of resistance, associated side effects, high cost, and challenges in reaching the target site, i.e., infected macrophages.^19^ For uncomplicated LCL, local therapy in the form of topical or intralesional administration is practiced routinely. Topical therapy is always superior to intralesional injections in terms of patient compliance, as there is no associated pain or anxiety. However, research data shows that the intralesional antimonials are more effective and have lower recurrence rates as compared to conventional topical creams.^20,21^

**Table 1:** Treatment Options for Cutaneous Leishmaniasis

**drugs effective for treatment of CL**	**Leishmania species**	**route of administration**	**dose**	**reference**
15% paromomycin/12% methylbenzethonium chloride ointment	all species	local therapy	Ointment application twice daily for 20 days	WHO,^22^
antimonials	all species	intralesional	1–5 mL per session every 3 to 7 days (1–5 sessions)	WHO
cryotherapy (liquid nitrogen: −195 °C)	all species	local application	1–5 sessions	WHO
thermotherapy (50 °C for 30 s)	all species	local application	1–2 sessions	WHO
fluconazole	*L. major*	oral	200 mg daily for 6 weeks	^23^
pentavalent antimonials	all species except *L. mexicana*	intramuscularly or intravenously	20 mg Sb^5+^/kg per day for 10 to 20 days and in case of L. aethiopica for 60 days to treat diffuse CL.	WHO
pentoxyfylline (combined with pentavalent antimonials)	*L. major*	oral	400 mg three times a day for 10–20 days	^24^
allopurinol (combined with pentavalent antimonials)	*L. tropica, L. b. panamensis*	oral	20 mg/kg for 30 days	WHO,^25^
ketoconazole	*L. mexicana*	oral	600 mg daily for 28 days	WHO,^26^
miltefosine	*L. guyanensis, L. panamensis, L. mexicana*	oral	2.5 mg/kg/day for 28 days	WHO,^9^
amphotericin B deoxycholate	*L. braziliensis*	I/V infusion	0.7 mg/kg/day for 25 to 30 doses	WHO
amphotericin B Liposomal	*L. braziliensis*	I/V infusion	3 mg/kg/day daily for 7 days and then twice weekly for further 3 weeks	^27^
imiquimod cream (combined with I/V meglumine antimoniate)	*L. braziliensis, L. peruviana*	topical application	5% cream every other day for 20 days	^28^

### Approaches to Improve Dermal Drug Accumulation

Major strategies that are commonly used to improve dermal drug accumulation include occlusive effects, supersaturation, the use of permeation enhancers, and novel drug delivery systems.^29^ Skin occlusion has long been used in dermatology to increase the penetration of topically applied medicaments and to improve wound healing. Occlusive dressing helps increase the retention time of ointments or other drug delivery systems on the skin and prevents early removal by protecting the skin against scratching and rubbing.^30^ However, the main reason for the increase in penetration of topically applied substances is shown to be an increase in the level of hydration of the stratum corneum (SC) under occlusion. This increased hydration disrupts the ultrastructure of the SC lipid barrier, thereby increasing the skin permeability.^31^

Various compounds from different chemical classes have been suggested as chemical penetration enhancers, including surfactants, lipids, and other hydrophilic and hydrophobic compounds (DMSO, propylene glycol, and ethanol). These compounds affect SC barrier properties and increase the permeability of active compounds through various mechanisms affecting both protein and lipid components of the stratum corneum.^32^ In contrast to previous methods to enhance permeability, supersaturation does not require alteration of the physicochemical properties of the skin barrier. In a supersaturated system, drug molecules are dispersed in a vehicle in amounts higher than their solubility, thereby imparting a high thermodynamic activity, which leads to enhanced skin permeability.^33^ However, these supersaturated systems may lead to drug crystallization over time because of thermodynamic instability.

Conventional drug delivery systems that are being used topically for the treatment of CL include creams, ointments, and hydrophilic gels. Application of nanotechnology to the field of topical drug delivery has provided new horizons to the treatment of skin disorders.^34^ Various types of nanoparticles have been used for improvement in drug delivery, either at dermal or transdermal levels.^35^ Figure 3 shows possible mechanisms by which lipid nanoparticles can target deeper skin layers and enhance skin localization. These novel drug delivery systems serve the purpose through various mechanisms, which include improvement in the solubility of poorly soluble drugs, enhanced skin penetration through SC, and provision of sustained drug release. These mechanisms account for an increased amount of the drug available at various skin layers and sustained drug release only at the target topical site, thereby minimizing systemic absorption and related side effects.^36^ Dermis targeting can be improved by surface modification with ligands or peptides that bind to skin-specific receptors or components. Various classes of nanoparticles that have been explored for their potential to improve drug delivery for the treatment of CL include lipid nanoparticles, such as liposomes, solid lipid nanoparticles (SLNs), nanostructured lipid carriers (NLCs), polymeric nanoparticles, and metallic nanoparticles, such as silver (Ag) and nanoparticles of metal oxides (titanium, silicon, and zinc oxides).^37^

**Figure 3:**
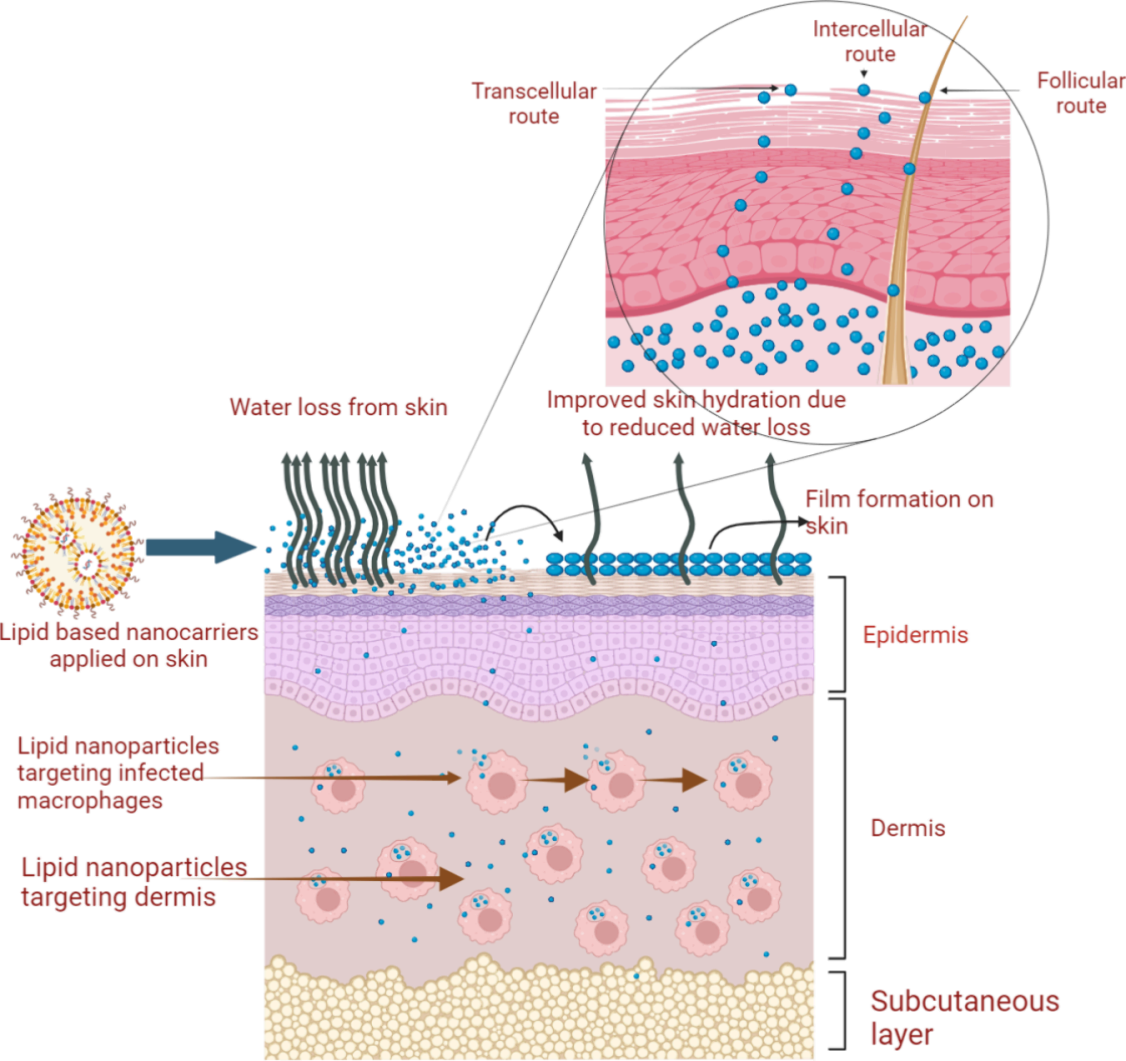
Lipid nanocarriers targeting dermis and macrophages (created in BioRender; Riaz, A. (2025) https://BioRender.com/r5f7vf8).

While other reviews have explored a variety of nanoparticles (metallic, polymeric, lipidic, etc.) in treating either visceral or cutaneous leishmaniasis,^38–40^ there is very limited literature available regarding a specific focus on lipid-based nanocarriers, especially in the context of topical applications. This review aims to fill that literature gap by concentrating on the efforts made for the development of novel lipid-based drug delivery systems for the delivery of antileishmanial agents for topical treatment of CL. First, we will discuss the general overview of various types of lipid-based nanocarriers and the possible advantages that these lipid-based nanosystems offer over other types of nanocarriers. We then discuss the possible mechanism of action of each class of lipid-based nanoparticles and their topical potential in detail. We then conclude with the application of lipid-based nanoparticles for the treatment of CL.

### Advantages of Lipid-Based Colloidal Carriers for Topical Application

Colloidal drug delivery systems have revolutionized the medical field since their introduction. They have been studied to overcome the problems associated with conventional systems, e.g., improved solubility of poorly soluble drugs, reduction in associated side effects, and improved therapeutic index.^41^ They are broadly categorized into two main categories, i.e., polymer-based and lipid-based colloidal systems, depending on the type of material used in their fabrication.^42^ The main advantages of lipid-based nanocarriers over other types of nanoparticles (polymeric, metallic) include the biocompatible and biodegradable nature of lipids,^43^ lower toxicity,^44^ loading of hydrophobic drugs,^45^ codelivery of hydrophobic and hydrophilic drugs,^46^ easy scale-up,^47^ enhanced flux across skin layers,^48^ and finally controlled drug release. Emulsion polymerization is a type of radical polymerization that usually starts with an emulsion incorporating water, a monomer, and a surfactant. It is the most common method of production for polymeric nanoparticles, which involves the use of potentially toxic cross-linkers and monomers that are difficult to remove completely at the end of the process.^49^ Another problem is the lack of cost-effective and suitable methods for the production of polymeric nanoparticles on a large scale. Lipid-based colloidal carriers were introduced mainly to overcome the toxicity issues of polymeric nanoparticles, as they are mainly composed of safe and well-tolerated excipients. Furthermore, their production can be undertaken on a large scale easily, which is a prerequisite for the successful entrance of any drug delivery system into the market. They can be produced on a large scale using various techniques: high-pressure homogenization, ultrasonication, spray drying, and coacervation. Research efforts have been oriented toward lipidic systems, including liposomes, nanoemulsions, and lipid nanoparticles (SLNs and NLCs), which will be described further in detail. Figure 4 represents each class of lipid nanoparticles that has been explored for dermal drug delivery of antileishmanial agents.

**Figure 4:**
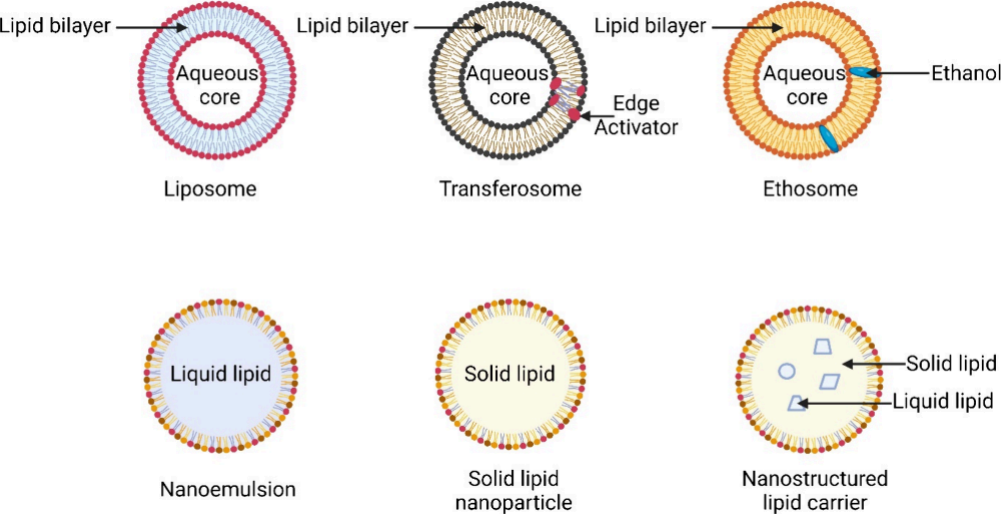
Liposome: lipid bilayer enclosing an aqueous shell. Transferosome: phospholipid nanocarrier containing single-chain surfactant inside the lipid bilayer, as edge activator. Ethosome: ethanol containing phospholipid nanocarrier. The globule of nanoemulsion: o/w type, oil globule stabilized by surfactant molecules. Solid lipid nanoparticle (SLN): solid lipid globule surrounded and stabilized by surfactant molecules. Nanostructured lipid carrier (NLC): globule consisting of a blend of solid and liquid lipid surrounded by surfactant molecules (created in BioRender; KHAN, M. (2025) https://BioRender.com/umarxxb).

### Liposomes

#### Potential of Liposomes in Topical Drug Delivery and Possible Mechanism of Action

Liposomes are nanostructured lipid vesicles that are composed of multiple lipid bilayers (mostly phospholipids) enclosing an aqueous shell.^50^ They provide many advantages when employed as a topical drug delivery vehicle, including their nontoxicity, biodegradability, and ability to encapsulate both hydrophilic and lipophilic drugs in the same package.^51^ Many methods have been reported in the literature for the production of liposomes, which are beyond the scope of this review, and readers may follow these references for details on manufacturing methods.^52^ Liposomes are especially significant for topical drug delivery because they can provide targeted drug delivery to various skin strata and reduce systemic drug absorption, therefore minimizing side effects. Modifying liposome size, surface charge, and chemical composition targets specific skin layers. Liposomes made of dipalmitoylphosphatidylcholine (DPPC) were among the first to be employed for topical drug delivery.^53^ Later, many researchers reported the ability of liposomes to localize in SC and deeper skin layers and suggested the usefulness of liposomes for dermal drug delivery.^54^

There may be many possible mechanisms that can explain enhanced cutaneous drug delivery when the drug is encapsulated in liposomes. The first concept focuses on the penetration of intact vesicles as a possible mechanism of enhanced skin accumulation. This hypothesis is supported by the fact that small unilamellar vesicles resulted in better accumulation of aqueous radiolabeled inulin in deeper skin layers as compared to larger-sized multilamellar vesicles, suggesting a role of particle size on skin deposition.^55^ Another mechanism suggested liposomes as penetration enhancers, in which liposomal lipids adhere to the skin surface, alter the lipid structure of SC, and penetrate the SC by disrupting and merging with the lipid matrix. This interaction depends on lipid composition, and the penetration-enhancing effect of liposomes has been demonstrated by many researchers. These lipid-based delivery systems can interact and fuse with the SC’s intercellular lipids. Because they are not as dense as SC, they can pass through sweat glands and hair follicles. They can be made to release the medication in a regulated way, which would lengthen the medication’s half-life in the skin and boost its effectiveness.^56,57^

#### Conventional Liposomes for Treatment of Cutaneous Leishmaniasis

All aforementioned properties of liposomes suggest them as a favorable candidate for the topical treatment of CL. Liposomal formulation of antileishmanial drugs (amphotericin B, stibogluconate, miltefosine, and paromomycin) has been used systemically to cure lesions of CL, which showed superior efficacy as compared to free drug or control groups.^58^ One group of researchers showed the potential of liposomes containing meglumine antimoniate (MA) to be taken up by macrophages infected with L. major more effectively as compared to free MA. Results of *in vitro* antileishmanial assay against amastigotes demonstrated that the MA-loaded liposomes resulted in lower IC_50_ values and were ≥ 10-fold more effective compared to free MA. The results of the macrophage uptake study, as determined by fluorescence microscopy, demonstrated that fluorescently labeled liposomes were taken up by infected macrophages (>80%) only after 10 min of incubation, with 100% of the cells showing fluorescence after 1 h. However, these studies demonstrated efficacy only *in vitro*, and further *in vivo* studies were suggested to confirm the efficacy of MA liposomes.^59^

A preclinical study demonstrated the efficacy of paromomycin sulfate (PM) loaded liposomes (LPMFs) in L. major-infected BALB/c mice.^60^ Liposomes containing different concentrations of paromomycin (10 and 15%) were synthesized by a fusion method. The skin permeation study for prepared formulations was conducted *in vitro* using mouse skin and a Franz diffusion cell, where results demonstrated that for both formulations, almost 60% of LPMFs were retained on the skin, and about 15% penetrated the skin. Topical application of LPMFs on lesions of infected BALB/c mice twice a day showed significantly smaller lesion size as compared to the control group, and a complete cure was achieved after 8 weeks. They proposed liposomes containing PM as promising carriers for the topical treatment of CL. The potential of PM-loaded liposomes for topical delivery was also evaluated by other researchers.^61^ Large multilamellar vesicles (MLVs) and large unilamellar vesicles (LUVs) were prepared by solvent evaporation and reverse-phase evaporation methods, respectively. Skin permeation experiments across normal and stripped skin demonstrated the controlled topical delivery of paromomycin for LUVs liposomes. Almost similar findings were reported in another study where liposomes enhanced the penetration of the drug across stripped skin *in vitro* and better antileishmanial activity in infected mice *in vivo.* Two types of liposomal vesicles were prepared using phosphatidylcholine (PC) alone or from a PC/cholesterol combination to encapsulate paromomycin (PA). The permeation of PM across intact skin was higher for liposomal formulations (7.2 ± 0.2% for PC and 4.8 ± 0.2% for PC/Chol) compared to the drug solution (1.9 ± 0.1%). The permeation of PM from liposomal formulations was also higher (20 times for PC and 10 times for PC/Chol) compared to the drug solution across the stripped skin. The results of the *in vivo* study on BALB/c mice infected with L. major also demonstrated superior efficacy of liposomal formulations compared to the drug solution in terms of relapse rate. By day 64, 50% of animals showed relapse in the PA solution-treated group, whereas the percentage of animals showing relapse in the liposomal formulation-treated group was only 10%. At the end of the study period, cure rates were demonstrated to be 0% for PA solution and 30% for liposomal PA.^62^

In yet another study, liposomes were used to encapsulate amphotericin B (AmB) for topical application to treat CL.^63^ In this study, two types of liposomes were prepared: liposomal AmB formulation and PEGylated liposomal AmB formulation. The efficacy of prepared formulations was evaluated both *in vitro* and *in vivo*, and results confirmed the drug-containing liposomal formulations to be more potent compared to standard AmB. Further, it was demonstrated that the addition of PEG in liposomes improved the antileishmanial efficacy *in vivo* in BALB/c mice in reducing the parasite burden by 1.6 fold and lesion size by 1.7 fold compared to nonPEGylated liposomal formulations. Compared to free AmB, the PEGylated liposomal AmB formulation demonstrated a decrease in lesion size by 5.9-fold and parasite burden by 2.5-fold. This improved efficacy of PEGylated liposomes was attributed to the smaller size compared to nonPEGylated liposomes, as smaller particles penetrate the target cells more efficiently.

Another possibility that can improve the efficacy of drug-loaded liposomes is the active targeting of nanocarriers to the diseased site. Most of the research in the area of treatment of CL using nanomedicines has utilized the passive targeting of nanocarriers to infected macrophages, especially in the case of topical drug delivery.^64^ Active targeting involves the surface modification of lipid nanocarriers using various ligands or antibodies that can specifically recognize the receptors on the target cells and thus enhance the uptake. Macrophages, the main target in the topical treatment of CL, contain many receptors that can be targeted to enhance the uptake of nanocarriers, including opsonic receptors (CD11b & CD44). Hyaluronic acid (HA), a glycosaminoglycan naturally produced in our body, can be recognized by CD44 and other HA receptors in macrophages.^65^ In one attempt, liposomes were coated with hyaluronic acid (HA), and when compared with noncoated liposomes, the former improved the antileishmanial activity of the entrapped active ingredient. These HA-containing liposomes were also able to penetrate deeper skin layers when applied topically and were proposed as a potential topical drug delivery system for the treatment of CL. The results of the skin permeation study demonstrated higher retention of loaded drug in the epidermis (0.69 ± 0.05 and 0.23 ± 0.07 for surface-coated liposomes compared to controls, where no drug could be quantified in deeper skin layers).^66^ In another study, the surface of cationic liposomes was modified with covalently attached p-aminophenyl-α-D-mannoside for the delivery of AmB. The results of the *in vivo* study in golden hamsters infected by L. donovani demonstrated superior antileishmanial activity in the case of mannose-coupled cationic liposomes, where a maximum reduction in the parasite load was observed compared to liposomes without any surface modification. The parasite burden was reduced by 42.5 ± 1.8, 61.2 ± 3.2, and 78.8 ± 3.9% in the case of AmB solution, AmB-loaded cationic liposomes, and AmB-loaded mannose-coupled liposomes, respectively.^67^ Overall, the active targeting of nanocarriers to infected macrophages seems to be a promising new area that requires further exploration, as limited research articles are available on this topic.

#### Ultradeformable Liposomes in Treatment of CL

To further improve the skin permeation properties, the next generation of conventional liposomes was developed, which have some useful properties like flexibility and ultradeformability. These include transfersomes and ethosomes that incorporate some useful alterations in the composition of traditional liposomes. Transfersomes are composed of phospholipids and contain single-chain surfactants as edge activators (tween, span, and sodium cholate), which destabilize vesicular lipid bilayers and make them highly deformable and elastic. This feature enables Transfersomes to squeeze through intercellular regions of the SC and improve skin permeation properties. They were reported to penetrate the skin *in vivo* with an efficacy comparable to subcutaneous administration. In a recent study, trifluralin-loaded transferosomes were evaluated for their targeted delivery to dermal skin layers and macrophages. Prepared drug-loaded transferosomes demonstrated superior *in vitro* efficacy against L. tropica promastigotes (3.07 fold reduction in IC_50_) and amastigotes (2.86 fold reduction in IC_50_) as well as a higher macrophage penetration compared to the drug solution. The results of this study demonstrated transferosomes as suitable candidates for cutaneous applications.^68^

Ethosomes contain phospholipids plus a high concentration (20–50%) of ethanol, which is a well-known permeation enhancer. The incorporation of ethanol provides the vesicles with a soft, flexible, and fluid nature, which allows them to penetrate deeper skin layers. Nanoethosomal formulations of amphotericin B were prepared by the mechanical dispersion method for the treatment of fungal skin infections.^69^ They proposed a nanoethosomal formulation as a promising carrier for topical skin delivery of AmB. Similarly, AmB-loaded ultradeformable liposomes were developed for the treatment of cutaneous fungal and leishmanial infections.^70^ These ultradeformable liposomes showed 75% and 100% L. braziliensis amastigotes and promastigotes activity, respectively, at a concentration of 1.25 μg/mL. A 40 times higher accumulation of the drug in human skin was reported for the prepared ultradeformable liposomal formulation as compared to Ambisome after 1 h of incubation in the absence of any occlusion. AmBisome is a marketed liposomal formulation of amphotericin B, which is antileishmanial and an antifungal drug. Figure 5 compares the structure of liposomes and ultradeformable liposomes as well as the possible mechanism of skin permeation for ultradeformable liposomes.

**Figure 5:**
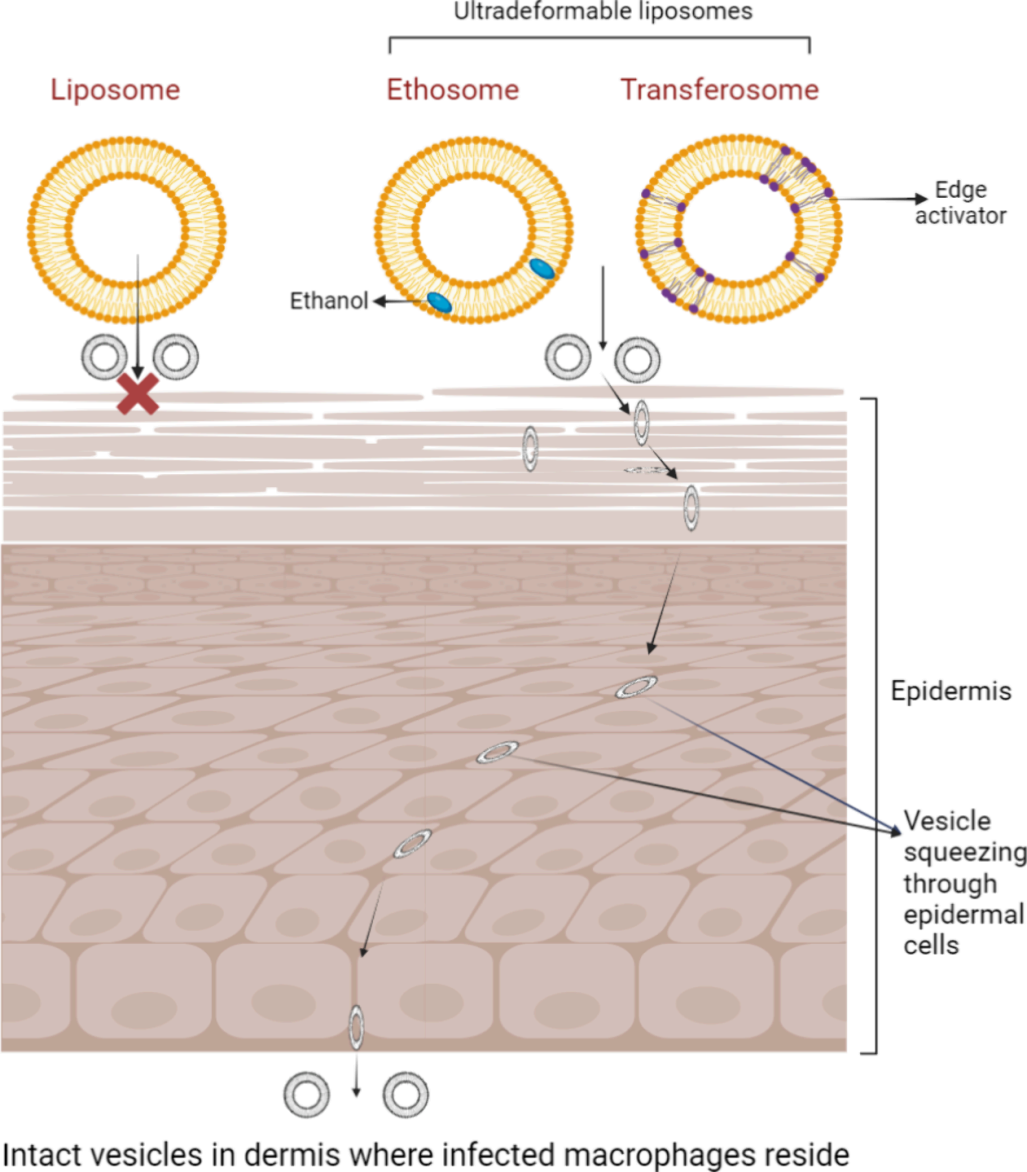
Structure of liposomes, transferosomes, and ethosomes (left) and skin permeation mechanism for ultradeformable liposomes (right) (created in BioRender; Riaz, A. (2025) https://BioRender.com/t63tl3h).

### Lipid Nanoparticles

#### Potential of Lipid Nanoparticles in Topical Drug Delivery and Possible Mechanism of Action

Both Solid lipid nanoparticles (SLNs) and nanostructured lipid carriers (NLCs) have numerous potential features that are valuable for dermal drug applications. First, they are colloidal drug carriers that provide sustained drug release at the site of application, and second, they are composed of biological lipids that ensure excellent tolerability and safety. Their small size results in enhanced contact with SC, which increases the amount of drug that penetrates into the skin layers. However, the most important property of lipid nanoparticles that provides improved drug accumulation in the skin is their potential to cause skin occlusion. Occlusion causes increased skin hydration due to reduced water loss from the skin, as described earlier.^30^

The occlusive potential of lipid nanoparticles of various sizes was studied at *in vitro, ex vivo, and in vivo* levels.^71^ Formulation based on SLNs resulted in thicker SC, and it has the potential to be used as an effective skin moisturizer. SLN-based formulations demonstrated excellent skin tolerability and were proposed as promising dermal drug delivery systems.^72^ Another interesting feature of lipid nanoparticles is their ability to target different skin strata. Various researchers have shown the potential of lipid nanoparticles to target skin layers, which is particularly beneficial for topical drug delivery in the treatment of different skin conditions. Skin targeting improves drug accumulation in specific layers and at the same time avoids excessive drug absorption into the blood.^73,74^ Another interesting study^75^ prepared tacrolimus-loaded lipid nanoparticles (T-LN) for the treatment of atopic dermatitis (AD). These nanoparticles were able to provide targeting and increased drug accumulation at epidermal and dermal layers, where immune-inflammatory cells responsible for AD are present. *In vitro* drug release and skin permeation properties were investigated using pig ear skin and compared with those of the marketed ointment. Confocal laser scanning microscopy (CLSM) was used to confirm the results of *in vivo* skin uptake. Results of the *in vivo* cutaneous uptake study demonstrated higher drug levels (3.36, 28.68, and 30.81 times in the stratum corneum, dermis, and epidermis, respectively) as compared to the available marketed ointment.

The mechanism of skin permeation and targeting of lipid nanoparticles largely remains unclear and requires a thorough investigation. However, it is clear that drug release from SLNs and NLCs largely depends on manufacturing conditions and process parameters. Various formulation parameters, such as lipid composition, lipid ratios, and manufacturing methods, can be finely tuned to achieve drug penetration and targeting control. Both burst release and sustained release can be obtained using lipid nanoparticles, while sustained drug delivery only in the skin strata for longer periods is most desirable for topical drugs. There may be many possible mechanisms for SLNs and NLCs to permeate the skin. For instance, intact lipid nanoparticles can permeate SC if they are of a very small size. However, in most cases, they accumulate in gland ducts, appendageal openings, and hair follicles of the skin, with smaller sizes showing higher accumulation. This would be ideal for treating cases of cutaneous Leishmaniasis where sustained therapeutic drug concentration is required in the dermis, which is the site of infected macrophages.^74,76^ In other cases, lipid nanoparticles simply act as a controlled drug delivery vehicle and remain on the outer skin surface, allowing only the drug to partition and permeate the skin. Another possible mechanism is that both drug and lipid molecules from SLNs permeate the skin independently and the interaction of skin lipids and lipids from SLNs forms a lipophilic reservoir region for lipophilic drugs, which will increase the residence time of such drugs in the skin.^77^

#### Lipid Nanoparticles for Treatment of Cutaneous Leishmaniasis

Although relatively less work has been done in the area of topical treatment of CL, both SLNs and NLC lipid nanoparticles showed promising enhancement in efficacy when antileishmanial drugs were loaded and tested against visceral Leishmaniasis.^78–81^ Many researchers prepared drug-loaded SLNs by conventional methods, and the results of these studies proposed that the prepared drug delivery systems have the potential to be used as a treatment for Leishmaniasis, owing to easy scale-up feasibility, low cost of fabricating materials, and efficacy compared to polymeric nanoparticles. However, these studies did not cover many aspects, such as *in vitro* and *in vivo* performance, and were unable to provide experimental evidence of efficacy against either VL or CL.^82^ Other studies provided proof of the *in vitro* efficacy of prepared lipid nanoparticle formulations against various Leishmanial strains and demonstrated a significant reduction in EC_50_ values compared to nonformulated drugs.^83,84^

Despite the tremendous potential of lipid nanoparticles for topical application and skin targeting for CL, very little work has been done in the area. Keeping this in view, our research group prepared topical drug-loaded NLCs-based gel to treat lesions of CL. Prepared drug-loaded NLC formulations showed significant targeting potential to deeper skin layers as well as macrophages.^85^ Encapsulation of amphotericin&nbsp; B (AmB) in NLCs demonstrated a significant reduction in IC_50_ values *in vitro,* against L. major promastigotes (105 μg/mL) and amastigotes (190 μg/mL), compared to free AmB (IC_50_ values of 243 μg/mL for amastigotes and 165 μg/mL for promastigotes). Finally, AmB-loaded NLCs demonstrated a significant drop in parasite load per mg of the lesion compared to free AmB and the control group when tested topically on L. major-infected mice.^86^ Our studies suggested NLCs as promising drug delivery carriers for topical treatment of skin conditions where targeting is required for deeper skin layers. Encouraging *in vitro* results motivated us to evaluate the potential of NLCs in treating lesions of CL *in vivo*. For this purpose, AmB-loaded NLCs were prepared, and efficacy was evaluated and compared to free AmB solution *in vitro* and *in vivo*. Results of *in vitro* antileishmanial assays demonstrated a significant reduction in IC_50_ values against promastigotes (0.02 ± 0.1 μM) and amastigotes (0.02 ± 0.1 μM) of L. major compared to free AmB solution (0.15 ± 0.2 μM for promastigotes and 0.14 ± 0.0 μM for amastigotes). Results of in vivo evaluation on BALB/c mice demonstrated that topically applied NLCs significantly reduced the parasite burden in the treatment group (65 × 10^8^ ± 13 per mg of the lesion) compared to control (>167.8 × 10^8^ ± 11 per mg of the lesion) and free AmB solution (>145.4 ± 18). These results demonstrated the topical potential of NLCs in treating the lesions of CL.^86^

### Lipidic Nanoemulsions

#### Potential of Nanoemulsions in Topical Drug Delivery

Nanoemulsions have tremendous potential in the topical delivery of medicaments owing to the following advantages: higher drug loading, lower skin toxicity, improved skin hydration, and permeation enhancement of drugs. Shakeel et al.^87,88^ conducted a study to gain insight into the skin permeation mechanism of a transdermally applied celecoxib nanoemulsion. Results of their experiments confirmed that nanoemulsion caused the extraction of lipids of SC, and significant disruption of the lipid bilayer was observed. Photomicrograph images showed voids and empty spaces in the epidermis caused by nanoemulsion application. Similar results were reported by the same researchers for the drug aceclofenac, where they proposed lipid extraction of SC as the main mechanism of drug permeation after the application of nanoemulsion.^89^

Amphotericin B-loaded nanoemulsion was synthesized as a cost-effective and efficacious alternative drug delivery system to liposomes for topical drug delivery.^90^ Different oil/water nanoemulsions were prepared, which contained varying concentrations of surfactant (Tween 80) and cosurfactant (PEG-400). These formulations were characterized, and skin permeation from optimized formulations (F-1, F–III, and F–VI) was compared with commercially available gel (Fungisome 0.1%). The *ex vivo* skin permeation study demonstrated enhanced skin permeation (1.85-fold higher than fungisome and 3.0-fold higher than the drug solution) and skin deposition (2.11-fold higher than fungisome) from the optimized nanoemulsion. They proposed the synthesized nanocarrier as a safe and economical topical treatment for fungal skin infections. Another study proposed nanoemulsion (NE) and nanoemulsion-based gel of amphotericin B (AmB-NE gel) for local skin delivery. All prepared formulations exhibited superior and statistically significant (*p* ≤ 0.001) antifungal activity as compared to reference drugs as well as a higher skin permeation flux rate in the case of NE (15.74 ± 0.4 μg/cm^2^/h) and AmB-NE gel (18.09 ± 0.6 μg/cm^2^/h) compared to free drug solution (4.59 ± 0.01 μg/cm^2^/h).^91^ Although these studies were not conducted on Leishmanial strains, these formulations have the potential to be used against CL owing to their skin-targeting potential in deeper layers.

#### Nanoemulsions for Treatment of CL

Chalcones are lipophilic compounds that have shown potential for the treatment of CL. Nanoemulsions containing synthetic chalcone were prepared and optimized for skin permeation and retention.^92^ Their results demonstrated that a formulation composed of polysorbate 20 and soybean lecithin showed good physicochemical properties and the highest dermal retention (3.03 μg g^–1^). Targeting and distribution into the dermis were also confirmed by confocal fluorescence microscopy. Results of the antileishmanial assay against strains of Leishmania amazonensis amastigotes proved that the optimized formulation retained antileishmanial activity after 60 days. They suggested soybean lecithin and polysorbate 20-based nanoemulsion as a suitable alternative for the topical treatment of CL. Some other studies also demonstrated the potential of microemulsion and nanoemulsion in the treatment of CL.^43,93,94^ However, these studies did not prove any superior *in vivo* efficacy, in contrast to marketed products. An interesting study where the topical potential of nanoemulsions was demonstrated *in vivo* to treat lesions of CL was undertaken by researchers.^95^ Nanoemulsion containing natural oils, extracted from P. emarginatus fruits (SO-NE), was prepared and employed in the topical treatment of CL. Results of the treatment on infected animals demonstrated a significant reduction of lesion sizes in the case of nanoemulsion (7.3 ± 0.8–5.6 ± 1.5 mm) compared to when oils were applied without formulation (6.7 ± 1.5–6.8 ± 1.6 mm). This study proposed topical nanoemulsion in combination with other standard treatments as a suitable drug delivery system for the topical treatment of CL.

### Other Lipid Nanocarriers for Topical Treatment of CL

Nanocochleates are another phospholipid-based system that is formed when negatively charged phospholipid bilayers are bridged using mostly calcium ions. These cigar-like nanostructures offer the advantages of increased stability, safety, and easy production. There are a few reports where nanocochleates have been used to improve the efficacy of antileishmanial drugs for the treatment of either visceral or cutaneous Leishmaniasis. The intralesional administration of nanocochleates containing essential oil from the plant Artemisia absinthium L. (EO-Aa-NC) demonstrated superior antileishmanial activity compared to control (nonformulated EO-Aa) by 50% in the BALB/c model of CL.^96^ The animals were allocated to five groups postinfection, with each group containing eight animals. One group did not receive any treatment, whereas the remaining four groups received intralesional EO-Aa, EO-Aa-NC, blank NC, and standard treatment Glucantime. Results of the *in vivo* study demonstrated that EO-Aa-NC was as effective as the standard drug Glucantime, whereas it also significantly reduced the lesion sizes (*p* < 0.05) compared to nonformulated EO-Aa and controls. Similarly, oral administration of amphotericin B-loaded nanocochleate formulation resulted in a higher accumulation of drugs in the organs (spleen and liver) of interest compared to the liposomal amphotericin B.^97^ There is limited literature that validates the topical potential of nanocochleates in treating skin diseases, although one report demonstrated the superior efficacy and stability of drug-loaded nanocochleates compared to liposomes for treating skin conditions.^98^ Given these encouraging results, nanocochleate formulations should also be investigated to enhance the antileishmanial efficacy when administered via the topical route for the treatment of CL.

Lipid-core nanocapsules (LNCs) are composed of a lipidic core that is surrounded by a polymeric envelope (usually biodegradable polycaprolactone). Encapsulating the active ingredients in LNCs confers protection from the external environment as well as provides a way to control the drug release.^99^ There are few reports of utilizing LNCs to improve the topical delivery of antileishmanial drugs. In one study, lipid-core nanocapsules were used to entrap antileishmanial chalcone (CH8) to analyze the topical potential of this nanocarrier in reducing the parasite burden in mice infected with L. amazonensis. Rhodamine-labeled LNCs were also prepared (Rho-LNC–CH8) to study macrophage uptake, and results demonstrated that Rho-LNC–CH8 were internalized by infected macrophages within 15 min. Results demonstrated that after 3 weeks of topical application every day, LNCs containing CH8 significantly reduced the parasite burden (86%) compared to the free CH8, which was ineffective. This improved efficacy was attributed to the efficient permeation of LNCs into the mouse skin as well as macrophage targeting of lipidic nanocarriers.^100^ Another study demonstrated significantly enhanced antileishmanial efficacy of quercetin (Qc) when encapsulated in LNCs compared to free Qc, after oral administration in the BALB/c mice infected with L. amazonensis. The results from these studies demonstrate the potential of LNCs that should be explored further in the topical therapy of CL.

### Controversies in Skin Permeation and Deposition of Lipid-Based Nanocarriers

The exact mechanism of skin permeation and deposition of lipid-based nanocarriers is still not clear and requires further investigation. Many studies reported the effect of particle size, composition, and surfactant type on skin permeation and localization potential, with smaller sizes showing better penetration compared to larger ones.^101^ Stratum corneum, being negatively charged, acts as a barrier to the passage of the anionic nanocarriers. Conversely, cationic nanocarriers will interact and accumulate, preferably in superficial layers. Therefore, neutral lipids are preferred to enhance skin permeation and deposition.^102^ The particle size of nanocarriers also depends on surfactant type, with certain surfactants producing smaller sizes compared to others.^103^ However, the exact mechanism behind this effect remains unclear and needs further investigation. Some conflicting evidence suggests that these nanocarriers largely remain in superficial skin layers and mainly act as reservoirs of loaded drugs to provide a sustained release rather than penetration to deeper layers^35^ To address these inconsistencies, Standardized methodologies in conducting skin permeation/deposition studies, both in vitro and in vivo, and advanced imaging techniques to accurately assess skin penetration are required.

### Safety and Efficacy of Lipid-Based Nanocarriers for Topical Application: Clinical Evidence

There are a limited number of clinical studies available on the topical application of lipid-based nanocarriers in the treatment of CL lesions. Table 2 summarizes the study design, lipid-nanocarrier-based formulation type, and key findings of available clinical trials specifically conducted to evaluate the safety and efficacy of lipid-based nanoformulations in topical drug delivery to treat CL lesions.

**Table 2:** Clinical Studies for Lipid-Based Nanocarriers in Topical Therapy of CL

**sr. no.**	**study design**	**disease treated**	**formulation**	**intervention**	**comparator**	**total enrolled patients**	**key findings**	**ref**
1	phase 3, randomized controlled trial	anthroponotic CL	nanoliposomes containing amphotericin B	topical liposomal AmB (0.4%) combined with meglumine antimoniate intralesional injection	meglumine antimoniate intralesional injection	130	the trial’s protocol, which is well-designed and approved by regulatory authorities, has been published; however, the trial’s final results are pending	^104^
2	retrospective study to evaluate safety and efficacy	lesions caused by *L. tropica* and *L. major*	nanoliposomes containing amphotericin B	topical nano liposomal AmB (0.4%) alone	topical nano liposomal AmB combined with intralesional Glucantime and/or cryotherapy	278	nnoliposomal AmB in combination with intralesional injections is a safe and effective approach to treat the lesions; 100% of patients demonstrated epithelialization of the wound, and recurrence was observed in only 2 patients out of 278; further field studies are recommended for the topical use of nanoliposomal AmB alone	^105^
3	open, pilot clinical trial to evaluate safety and efficacy	lesions caused by *L. major*	liposomes containing AmB	topical liposomal AmB (0.4%) alone	topical liposomal AmB combined with intralesional meglumine antimoniate	66	topical liposomal AmB, whether alone (95% cure rate) or in combination (92% cure rate), demonstrated good safety and acceptable efficacy	^106^
4	randomized, double-blind, phase I clinical trial	NA	nanoliposomal formulation of AmB	topical application of liposomal formulation twice a day for 7 days	topical application of the same formulation 3 times a day for 14 days	27	topical application twice a day demonstrated good safety with no adverse effects; however, skin reactions were reported in the other group with thrice daily application; the liposomal formulation was safe to use, and phase II trials can be conducted using twice daily application	^107^
5	randomized, placebo-controlled, double-blind trial	lesions caused by *L. major*	liposomal AmB gel	topical application of liposomal AmB 4% gel twice daily for 28 days followed by a second round for an additional 28 days	topical application of placebo gel twice daily followed by the next round where liposomal AmB gel was applied twice daily for 28 days	13	treatment was well tolerated with no serious side effects, whereas clinical improvements were noted in both groups; a reduction in swelling, ulceration, and induration was more significant on day 56 compared to day 28	^108^
6	randomized, open-label trial	lesions of CL (old world)	liposomal azithromycin	topical twice-daily application of liposomal azithromycin (30 mg/mL) combined with oral azithromycin tablet (250 mg) twice daily	oral azithromycin therapy alone	27	no serious adverse effects were reported and the combination group demonstrated significant improvement in lesion size and induration after 12 weeks compared to only orally administered azithromycin; combination therapy is a safe and effective alternative to treat CL.	^109^
7	randomized, open-label, and comparative clinical trial	lesions of CL	liposomal azithromycin	topical liposomal azithromycin twice daily	intralesional Glucantime injection	66	therapy was well tolerated by both groups, and no major adverse effects were reported; topical liposomal azithromycin demonstrated similar efficacy to intralesional Glucantime with no significant difference between the two groups in terms of outcomes (*p* = 0.67)	^110^
8	randomized, double-blinded clinical trial	lesions of CL	liposomal clarithromycin	topical application of liposomal clarithromycin lotion (1%) combined with intralesional Glucantime	topical application of normal saline as a placebo combined with intralesional Glucantime	60	no adverse events were reported in both cases; lesions sizes, in the first and last follow-ups, were observed to be significantly smaller in the clarithromycin group (7.73 ± 4.31 to 0.48 ± 0.50) compared to Glucantime only group (5.47 ± 5.83 to 0.76 ± 0.88) and there was no significant difference among both groups in terms of recovery time.	^111^

Currently, the lesions of CL are treated with either intralesional injections or systemic administration of drugs including liposomal AmB as presented in Table 1. Topical therapy is used conventionally as an adjunct to systemic therapy because topical therapy with traditional formulations alone is unable to produce a satisfactory clinical response due to several challenges mentioned earlier in the manuscript. Lipid-based nanocarriers have demonstrated promising results for topical application, either alone or in combination with intralesional therapy, both *in vitro* and at the preclinical level. Among lipid-based nanocarriers, liposomal formulations are already approved for systemic administration to treat both visceral and CL. However, clinical trials specifically for topical application for treating the lesions of CL were only found for liposomes and are summarized in Table 2. Results from these clinical trials demonstrated that topical application of liposomes containing antileishmanial drugs, whether alone or in combination with standard therapies, was safe and highly effective in reducing the lesion size and severity of ulceration and induration. One study demonstrated that topical azithromycin alone produced results comparable to those of intralesional glucantime (standard therapy), highlighting the potential of topical application that can be used as a standalone treatment for self-limiting cases of CL, eliminating the need for systemic therapy. In another study, combining topical application with oral therapy produced better results than oral therapy alone. This suggests the potential of topical application to complement the standard therapies that can reduce recovery time and hospital visits while potentially minimizing the need for hospitalization in severe cases of the disease.

Lipid nanoparticles, on the other hand, have found their way into the market to deliver vaccines, including the COVID-19 vaccine. Several clinical trials are underway for the delivery of mRNA-based vaccines for various cancers and solid tumors using lipid nanoparticles.^112^ There are a few reported clinical trials for the topical application of lipid nanoparticles-based formulations to treat various skin conditions, including acne vulgaris and pityriasis versicolor.^113,114^ The results of these trials demonstrated the superior efficacy of SLNs-based formulation compared to the commercial product in treating pityriasis versicolor, whereas in the case of acne vulgaris, the formulation based on NLCs provided skincare benefits as well. Similarly, the topical potential of nanoemulsions (NE) was also confirmed by a few clinical studies. For example, in one study, NE containing adapalene proved to be superior in terms of efficacy compared to marketed adapalene gel to treat acne vulgaris.^115^ However, clinical trials that evaluate the topical potential of lipid nanocarriers, other than liposomes, in treating the lesions of CL are missing and highlight a research gap that needs addressing.

## Challenges and Future Perspectives

Lipid-based nanocarriers (LNCs) also face numerous challenges in the treatment of CL. One of the key obstacles in topical drug delivery systems is the stratum corneum. It acts as a protective barrier and limits drug penetration. Although lipid-based nanocarriers are designed to enhance transdermal absorption, skin properties, lipid composition, and hydrophilicity variations can lead to inconsistent drug delivery. Moreover, there also remains uncertainty regarding nanoparticle penetration and their ability to reach infected macrophages.^2^ Apart from that, LNCs face stability issues such as degradation, drug leakage, and phase separation during storage. Physicochemical properties might be altered due to temperature fluctuations and exposure to external environmental factors, thus reducing their therapeutic efficacy over time.^12^ Another major challenge is to maintain an optimal balance between the drug encapsulation efficiency and controlled drug release. Repeated application of nanocarriers with surfactants or penetration enhancers may lead to local irritation or hypersensitivity reactions, raising concerns about their prolonged use in CL therapy.^37^ Large-scale manufacturing of lipid-based nanocarriers is cost-intensive because it requires precise control over particle size, charge, and encapsulation efficiency. The transition from laboratory-scale research to commercial-scale production remains a bottleneck due to variability, a lack of standardization, and complex formulation methods. Regulatory guidelines for nanomedicine approval are still in evolving stages, and the lack of specific criteria for lipid-based nanoformulations poses additional challenges.^116^

Future research should focus on the aforementioned key areas to overcome these challenges and advance the clinical application of lipid-based nanocarriers for the treatment of CL. For this purpose, nanocarrier modifications can be explored to optimize drug penetration across the skin barrier. Ligand-functionalized nanocarriers, microneedle-assisted drug delivery systems, and hybrid nanoparticle systems combining lipid-based carriers with polymeric nanostructures may enhance penetration and drug retention time.^117^ Development of lyophilized nanoformulations with cryoprotectants can significantly increase storage stability and prolong shelf life. Microfluidic-based nanocarrier synthesis is another promising approach for large-scale and reproducible production by ensuring precise control over particle size, charge, and encapsulation efficiency.^118^ Another focus should be on conducting long-term *in vivo* safety studies to assess the immune response, potential skin irritation, hypersensitivity reactions, and systemic toxicity to repeated applications of lipid-based nanocarriers. Furthermore, lipid formulations with natural phospholipids, sterols, and antioxidant-loaded carriers can be explored to minimize toxicity. Future studies should also conduct randomized clinical trials to establish the efficacy, safety, and patient acceptability of lipid-based nanocarriers for CL. Finally, there is also a dire need to establish standardized characterization protocols for LNCs in dermatological applications. Regulatory agencies (e.g., FDA, EMA, WHO) can play a crucial role in defining acceptable toxicity limits, stability criteria, and manufacturing guidelines.

### Toxicity Considerations of Lipid-based Nanocarriers

Although the use of lipidic nanocarriers has gained widespread attention from researchers in drug delivery, there is still a limited set of data that specifically explores the toxic effects of these nanocarriers on healthy and/or diseased tissues and cells. The possible immunological reactions that these nanoparticles can elicit are another lesser-known area and need further research so that these systems can be improved and enter the market. In the case of lipidic nanocarriers, toxicities and immune responses can arise from the lipid components as well as various other excipients (surfactants, cholesterol, etc.) and compounds used for surface modifications (PEG, conjugated antibodies).^119^

Cationic liposomes enhance the stability and delivery of negatively charged nucleic acids and therefore are being used extensively for said purpose. Many researchers have reported the toxic effects of cationic liposomes on various cells, especially macrophages and monocytes. Cationic liposomes were labeled as highly toxic to macrophages with cytotoxic potential increased at higher zeta potential values.^119,120^ Interestingly, in the case of topical delivery in treating CL, this higher cytotoxicity toward macrophages can be useful in combating the infected macrophages. The presence of cholesterol in liposomes, although it has added benefits as previously discussed, can impart independent toxicity problems as well as release of inflammatory mediators (TNFα- & IL-6).^121^ There are also reports of hypersensitivity reactions after the administration of liposomes that are attributed to complement activation due to the surface PEG of stealth liposomes.^122^

On the other hand, SLNs and NLCs are composed of lipids that are generally recognized as safe (GRAS) excipients and are considered safe.^123^ However, newer research on these lipid nanoparticles involves surface modification to actively target the drugs, which requires the use of various linking agents.^124^ These linkers and cryoprotectants can elicit immune responses and toxicity problems.^125,126^ To demonstrate the biocompatibility of lipid nanoparticles in vitro, researchers used cell viability assays on various cell lines. These cell lines can tolerate SLNS or NLCs in doses up to 1–2 mg/mL.^127,128^ Selection of a suitable surfactant is also crucial to developing a biocompatible lipid nanoparticle formulation in which nonionic surfactants are always preferable. However, if the application requires the use of a cationic surfactant, it always comes with a risk of decreased compatibility. Cationic surfactants can cause immune system sensitization as well as create pores and defects in the cell membranes.^129,130^ There are also numerous reports of evaluating skin irritation potential using animal models that demonstrate these lipid nanoparticles as extremely safe topical systems with either no signs of irritation to the skin (edema or erythema)^131,132^ or significantly less irritation compared to the marketed formulation of active ingredient.^133,134^

Nanoemulsions are mainly composed of ingredients that are considered safe and well tolerated. However, the main component that can cause toxicity is the surfactant (cationic, anionic, or nonionic). In addition, nanoemulsions can be formulated to contain various other ingredients like gelling agents, ripening inhibitors, cosurfactants, or organic solvents that can indirectly impart toxicity by altering cell membrane permeability.^135^ There was no significant difference in the toxicity of nanoemulsions when compared to emulsions using Caco-2 cell lines, which shows that a reduction in particle size did not affect toxicity.^136^

In the case of topical therapy, the main purpose of the formulation is to reach into the skin layers while avoiding systemic uptake. Therefore, the systemic toxicity that may arise after the topical application of nanoformulations must be taken into account. Although lipidic nanocarriers mainly comprise well-tolerated and safe lipids, the effect of other excipients should always be evaluated when nanotoxicology. Undesired immunological effects and hypersensitivity reactions are some other aspects that can result in reduced safety of lipid nanocarriers, and more research work is needed in this area to produce fully capable topical drug delivery systems.^137,138^

Each class of lipid-based nanocarriers has its specific advantages and concerns regarding topical application. Liposomes and ultradeformable liposomes can squeeze through the stratum corneum barrier and provide enhanced skin flux, while at the same time, they possess stability issues that require postprocessing steps, such as freeze-drying, to enhance the stability of the final formulation. Lipid nanoparticles have improved stability, and there is the possibility of topical gel formulations that can be stable over a storage period, but there is the issue of drugs leaking out of the nanocarriers over time. This problem was overcome by the introduction of nanostructured lipid carriers. Nanoemulsions have also been traditionally linked to drug leaking, highlighting the area for further research to improve their stability.

## Conclusions

Topical delivery is an attractive alternative treatment route for cutaneous leishmaniasis (CL), offering significant advantages over systemic therapy. This review demonstrated the potential of lipid-based colloidal carriers in enhancing topical drug delivery, particularly for CL. These nanocarriers provide key benefits such as improved skin penetration, occlusive effects, enhanced skin targeting and deposition, as well as sustained drug release at the target site, addressing some of the challenges of conventional dosage forms. Both *in vitro* and *in vivo* studies have consistently demonstrated promising results in improving topical drug efficacy to treat the lesions of CL.

Despite these advantages, challenges remain, including formulation optimization, large-scale production, and clinical validation. Even with encouraging preclinical findings in the field, there is a limited set of data on clinical trials, and that too is confined to liposomes. Future research should focus on refining the formulation parameters, improving the stability, and conducting large-scale extensive clinical trials to facilitate regulatory approval and real-world application. The use of advanced production techniques, such as microfluidics, can further improve the reproducibility and scalability of lipid-based nanocarriers. Given that liposomes and lipid nanoparticles are already in commercial use for pharmaceutical and cosmetic applications, their transition to CL therapy is promising. Overcoming these remaining barriers will pave the way for lipid-based nanocarriers to become a mainstream therapeutic option, offering a safer, more effective, and patient-friendly treatment for CL.
